# Pilot Trial of Neuromuscular Stimulation in Human Subjects with Chronic Venous Disease

**DOI:** 10.2147/VHRM.S320883

**Published:** 2021-12-01

**Authors:** Katherine J Williams, Hayley M Moore, Mary Ellis, Alun H Davies

**Affiliations:** 1 Academic Section of Vascular Surgery, Imperial College London, London, UK

**Keywords:** neuromuscular, electrical, stimulation, venous disease, venous insufficiency

## Abstract

**Introduction:**

Neuromuscular stimulation (NMES) has been shown to improve peripheral blood flow in healthy people. We investigated the effect of bilateral leg NMES on the symptoms of chronic venous disease.

**Methods:**

Forty subjects were recruited from four groups: healthy, superficial insufficiency, deep insufficiency, and deep obstruction. Haemodynamic venous measurements were taken from the right femoral vein with ultrasound, laser Doppler fluximetry from the left hand and foot. Devices were then worn for 4–6 hours per day, for 6 weeks. Haemodynamic measurements were repeated at week 6. Quality of life questionnaires were taken at week 0, 6 and 8.

**Results:**

The mean age was 48.7, BMI 28.6kg/m2, and maximum calf circumference 39.0 cm. Twenty-four subjects were men. NMES increased femoral vein peak velocity, TAMV and volume flow by 55%, 20%, 36% at 20 minutes (all p<0.05), which was enhanced at week 6 (PV and TAMV p<0.05). Mean increases in arm and leg fluximetry were 71% and 194% (both p<0.01). Leg swelling was reduced by mean 252.7 mL (13%, p<0.05) overall; 338.9 mL (16%, p<0.05) in venous disease. For those with venous pathology, scores for disease specific and generic quality of life questionnaires improved. Those with C4-6 disease benefitted the most, with improvements in VDS score of 1, AVVQ of 6, and SF-12 of 10.

**Conclusion:**

NMES improves venous haemodynamic parameters in chronic venous disease, which is enhanced by regular use. NMES reduces leg oedema, improves blood supply to the skin of the foot, and may positively affect quality of life.

**Clinical Trials:**

This trial was registered with www.clinicaltrials.org.uk (NCT02137499).

## Introduction

Venous disease is common, and increasing levels of prevalence have been observed.^1^,^2^ The incidence of superficial venous disease is roughly 40–55% in Europe, and around half of these will have symptomatic varicose veins.^3^ The Bonn Vein Study estimates the prevalence of symptomatic venous disease to be ~32%.^4^ Established venous dysfunction of any aetiology (chronic venous disease: CVD) is due to the inability of damaged veins or venous valves to effectively return blood to the heart from the lower extremities. The consequent venous hypertension and stasis contribute to clinical symptoms, and long-term complications, such as venous ulcers are a significant health burden.^5^,^6^ Standard treatments aim to reduce venous stasis by improving venous return. Surgery can ablate dysfunctional vessels, whilst compression hosiery or layered bandaging aids the deep compressive ability of contracting muscles. Extrinsic compression of the leg can be achieved with intermittent pneumatic compression (IPC), and has been shown to be effective in the treatment of CVD.^7–9^

Venous thromboembolism is a common and preventable cause of morbidity and mortality, affecting 1 in 1000 adults per year.^10^ It is the most common cardiovascular cause of death following coronary artery disease and stroke.^11^ The incidence rates of deep vein thrombosis and pulmonary emboli are approximately 148 per 100,000 person-years and 95 per 100,000 person years, respectively.^12^ The prevention of deep venous thrombosis is an important clinical issue in the context of immobility, dehydration, systemic illness, the peri-operative period or pro-thrombotic state. Prophylactic measures either reduce coagulability (anticoagulant drug therapy), or reduce stasis (external mechanical interventions).^13^,^14^

Activation of the venous pump muscles themselves can be achieved through electrical stimulation of the nerves and muscles in the leg, bypassing normal active mechanisms. This allows muscle pump activity with the subject at rest: useful for periods of inactivity, exercise recovery, or peri-operative care. Neuromuscular electrical stimulation (NMES) has been used by clinicians both in the prevention of venous thrombosis and the management of circulatory problems, and has been shown to have an equivalent haemodynamic effect to IPC in healthy subjects.^15–19^ Medically licensed devices are available on the open market, and are portable, easy to use, and relatively inexpensive.

## Aims

In this pilot trial, the role of NMES in both healthy subjects and patients with CVD has been evaluated, testing the hypothesis that repeated NMES use will enhance venous haemodynamic parameters, improve clinician disease scores, and increase patient-reported quality of life.

## Methods

This study was conducted in accordance with the declaration of Helsinki. Ethical approval for a controlled interventional trial was obtained from National Research Ethics Committee (13/WM/0027 – “The VeINS Trial”). All the subjects were over 18 years old. Subjects were excluded if they had a history of peripheral arterial disease, leg fracture or metallic implant, or other systemic causes of limb swelling (heart, lung or renal failure).

To evaluate the effect of NMES on both healthy subjects, and patients with venous disease, we chose our study populations accordingly. Recruitment of venous patients was stratified according to patterns of disease reported by clinical vascular scientists using ultrasound (superficial vein incompetence, deep vein incompetence, deep vein obstruction), as this might affect haemodynamic performance with the NMES device. We did not analyse the associations between pattern and severity of disease.

All study baseline ultrasound examinations were performed within one week of recruitment. Healthy subjects (n=10) were recruited by word of mouth, and venous patients (n=30) from vascular clinic and the imaging laboratory. Written informed consent was recorded. Screening included a medical history, physical examination, pregnancy test (females), and ultrasound examination of the lower limbs.

Due to the paucity and heterogeneity of available literature on neuromuscular stimulation, we were unable to perform a power calculation, since effect size could not be estimated at this stage. Ten subjects in each of the groups were deemed an acceptable number for a feasibility trial.

Venous compression ultrasonography was performed on all recruits, with clinically significant reflux defined as greater than 0.5s.

Initial duplex ultrasound screening was performed with subject standing, using iU22 xMATRIX ultrasound system with L9-3 transducer and venous protocol (Philips, Seattle, WA, USA). Ankle-brachial pressure index (ABPI) was calculated for both lower limbs, with the lowest of the two values reported.

### The NMES Device

The NMES device used was the geko™ T-1 (Firstkind Ltd, UK). Applied transcutaneously to the common peroneal nerve at the knee, intermittent electrical pulses (27mA, 1 Hz) cause contractions of the anterior and lateral compartments of the leg. The minimum pulse width (70–560μs) to achieve dorsiflexion was used, and adjusted by the patient for comfort. There is no established protocol for this device in the treatment of venous disease. Given that it has been designed to be used as needed, we designed a protocol we thought to be both sensible and tenable. Devices were fitted bilaterally, and worn for 4–6 hours, 5 days a week, for 6 weeks. A usage diary was collected from each subject at the end of the testing period.

### Measurement of Haemodynamic Variables

Prior to haemodynamic assessment, subjects were allowed to quietly rest for 10 minutes, semi-recumbent on an examination couch. All measurements were made at a controlled room temperature of 22 degrees Celsius, by the same investigator. The right femoral vein was marked for repeat measurements, 3–5 cm from the saphenofemoral junction, as per Williams et al.^15^ Five repeat measurements of venous parameters – peak velocity (PV), time averaged mean velocity (TAMV), and volume flow (VF) – were taken from the femoral vein, and respective means were calculated for each parameter. Laser Doppler fluximetry (VMS-LDF2, Moor instruments, UK) was recorded from the dorsum of the left hand and foot. Coefficients of variation for fluximetry units and temperature were 0.7% and 0%, respectively (n=20, serial readings taken from dorsal pedal skin of a healthy individual at rest). Baseline measurements were taken before activation of both devices, and during the device activation, 20 minutes after the device was turned on and adjusted to effect. Percentage changes were derived as the differences between “device off” and “device on”. Haemodynamic measurements were repeated at six weeks with the same protocol.

Leg volume (V) was calculated by measuring ankle and maximal calf diameters with a tape measure, the vertical distance between the two points (h), deriving ankle and calf radii (R1 and R2), and applying a truncated inverted cone model.^20^ This was performed bilaterally.

$$ V = {{\pi h} \over 3}\left({R{1^2} + R1R2 + R{2^2}} \right)$$

### Six Week Device Usage

Participants were trained in how to apply the NMES device themselves and given a supply to use at home. The training involved a face-to-face meeting, demonstration of device skin application, how to turn the device on and off, and the desired muscle twitch to elicit. After week 6, NMES was discontinued. Physician- and patient-reported quality of life assessments (Venous Clinical Severity Score,^21^ Venous Disability Score, Aberdeen Varicose Vein Questionnaire, EuroQol-5D, Center for Epidemiologic Studies Depression (CESD) Scale,^22^ Short Form-12) were performed at weeks 0, 6, and 8. An unsupervised diary of device usage was completed by each participant.

### Analysis

Demographic and anthropometric data across groups were compared using one-way ANOVA. Haemodynamic parameters were measured as changes from baseline, and compared between healthy subjects and pooled patients with venous disease. Differences between venous disease groups were compared using quality of life scores, interpreted in the context of disease severity: grouped into CEAP C0-3 (mild venous disease) and C4-6 (severe venous disease). It was thought that this would reflect real-life clinical decision-making. Data were analysed using IBM SPSS Statistics version 21. The Kolmogorov–Smirnov test was used to assess the normality of the distribution, and then the appropriate parametric or non-parametric tests were applied. Parametric data are reported using mean and standard deviation. Non-parametric data are described using median and interquartile range. Results were considered significant at p<0.05. 

## Results

**Figure 1:**
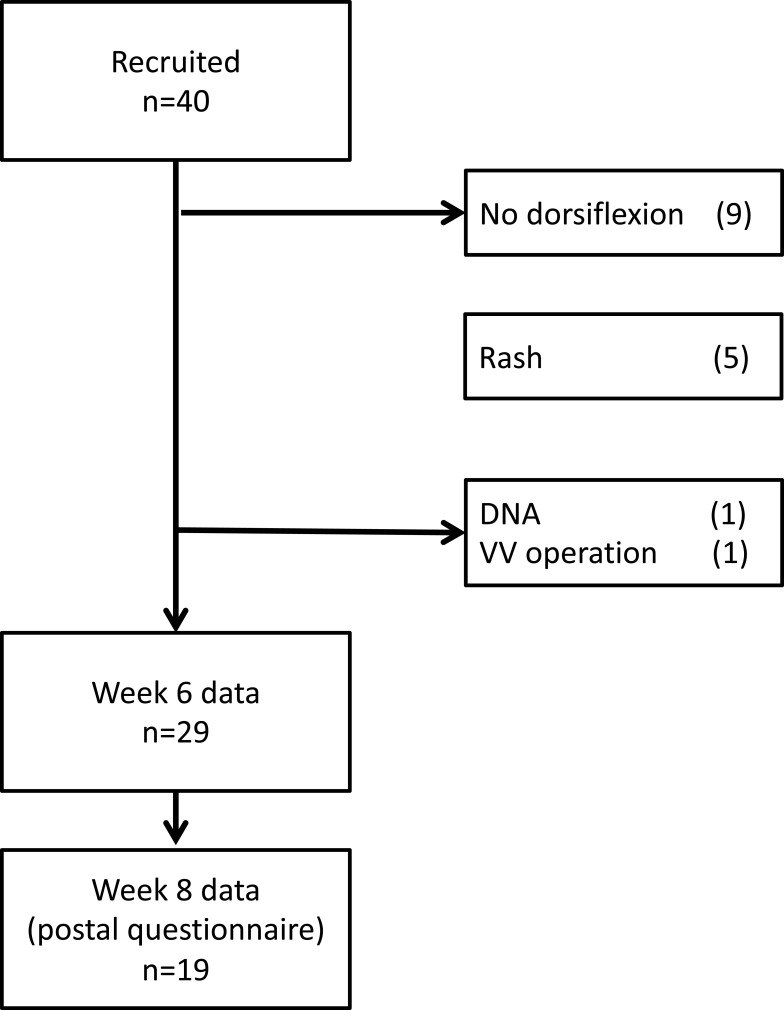
CONSORT diagram of VeINS trial (DNA – did not attend follow-up, VV – varicose vein).

Ten healthy subjects and thirty venous patients were recruited and grouped. There were 24 men (60%), mean age was 49.4±18 years, BMI 29.0±7kg/m^2^, calf circumference 40.4±6cm, and unilateral leg volume 1927.2±826mL. Five patients in group three had mixed deep and superficial diseases, and two patients in group four had mixed superficial and deep diseases. Demographic and clinical characteristics of each group are shown in Table 1, with anatomical segments affected shown in Table 2. Previous superficial venous surgery had been performed in 6 subjects in the superficial reflux group, and 5 in the deep reflux group. All surgery was more than 6 months old. Figure 1 shows the CONSORT diagram.

**Table 1:** Demographic Data of Recruited Subjects

	Healthy Subjects	Superficial Venous Insufficiency	Deep Venous Insufficiency	Deep Venous Obstruction	ANOVA
Number	10	10	10	10	
Age (years)	30.7±10	55.0±15	58.6±18	53.2±16	<0.001
Sex	50% M	40% M	80% M	70% M	0.04
BMI (kg/m^2^)	23.6±4	26.4±6	30.1±3	34.8±6	<0.001
CEAP clinical severity score	0.8±1	3.1±1	4.4±1	4.0±1	<0.001
Unilateral leg volume (mL)	1451.1±574	1730.2±390	2025.8±1029	2501.7±814	<0.001

**Note**: Mean ± standard deviation.

**Table 2:** Anatomical Segments Affected by Venous Disease in Trial Patients (Disease may affect more than one segment in any one subject, therefore total not equal to participant number in each group)

Vein Segment Affected	Superficial Venous Insufficiency	Deep Venous Insufficiency	Deep Venous Obstruction
Venae cava	0	0	1
Iliac	0	0	4
Femoral	7	9	6
Popliteal	6	8	1
Below knee (including perforators)	9	8	3

The NMES device was ineffective (unable to elicit a twitch) in nine patients, with a mean BMI of 35.5±5kg/m^2^, calf circumference 45.9±6cm, CEAP C4.1±0.6, and AVVQ 31.3±14.

### Haemodynamic Results

**Figure 2:**
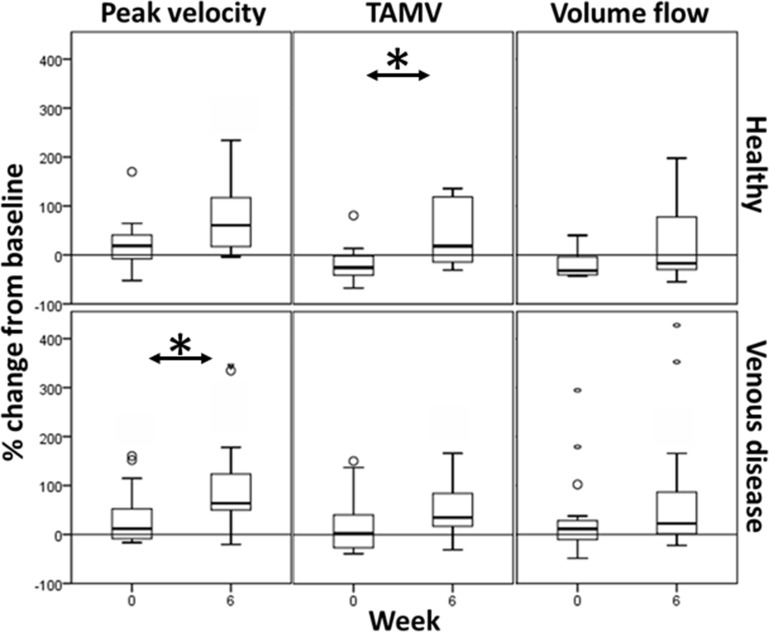
Haemodynamic changes in the femoral vein with neuromuscular electrical stimulation, taken at weeks 0 and 6 (*p<0.05, Wilcoxon matched pairs signed-rank).

Median changes with device activation in PV (peak velocity), TAMV (time averaged maximum velocity) and VF (volume flow) in healthy subjects were 34% (6.5cm/s, IQR −1 – 12, p=0.01, Wilcoxon signed-rank), −14% (−1.0mL/min, −2 – 2, p=0.9) and −23% (−29.7mL/min, −64 – 51, p=0.6), respectively. Median changes in PV, TAMV and volume flow in those with venous disease were 41% (3.4cm/s, IQR −1 – 13, p<0.001), 25% (0.4 cm/s, −1 – 2, p=0.02) and 20% (12.7mL/min, −17 – 51, p=0.01). Gains were less in subjects with deep venous disease (see Table 3). Regular use over 6 weeks enhanced all stimulation venous parameters in both health and disease, which was statistically significant for TAMV in healthy subjects, and PV in venous disease (Figure 2).

**Table 3:** Haemodynamic changes in the femoral vein, with use of NMES, “Device off” compared to “Device on”

Percentage Change from Baseline	Group							
	Healthy Subjects		Superficial Venous Insufficiency		Deep Venous Insufficiency		Deep Venous Obstruction	
	Median	IQR	Median	IQR	Median	IQR	Median	IQR
Peak velocity	34.8**	−4–81	62.8**	25–138	9.0	−10 – 84	14.8	−8–51
TAMV	−14.2	−30 – 55	28.1**	−2 – 111	28.2	−30 – 66	−5.1	−28–35
Volume Flow	−22.5	−40–40	37.5*	−10–172	17.4*	1–49	5.9	−11–21

**Notes**: *p<0.05, **p<0.01, Wilcoxon signed-rank.

**Abbreviation**: IQR, interquartile range.

Mean increases in fluximetry readings in healthy subjects' arms and legs are shown in Table 4. Increases in leg fluximetry signal were attenuated in deep venous disease. Changes in fluximetry signal with device activation were not affected by repeated use over 6 weeks.

**Table 4:** Laser Doppler Fluximetry changes in the hand and foot with use of NMES, “Device off” compared to “Device on”

Percentage Change from Baseline	Healthy Subjects	Superficial Venous Insufficiency	Deep Venous Insufficiency	Deep Venous Obstruction
Leg flux	274.8 ± 279**	264.9 ±283*	69.3 ± 126	46.9 ± 50*
Arm flux	84.0 ± 126**	71.3 ± 76*	23.7 ± 57	106.5 ± 115*

**Notes**: Mean ± standard deviation; *p<0.05, **p<0.01, Wilcoxon signed-rank.

### Leg Volume Changes

Over 6 weeks median unilateral leg volume decreased by 0.2% (26.3 mL, p=0.61) for healthy subjects, and 11.6% (225.4 mL, p=0.03) for those with symptomatic venous disease. This is shown in Table 5.

**Table 5:** Changes in unilateral leg volume over 6 weeks regular NMES use

	Healthy	Venous Disease
Week 0 (mL)	1451.1 ± 574	2085.9 ± 840
Week 6 (mL)	1552.2 ± 580	1797.2 ± 491
Significance (Wilcoxon signed-rank)	0.61	0.03

**Note**: Median ± interquartile range.

### Quality of Life Measurements

For reporting reasons with a small number of subjects, venous severity results were grouped according to CEAP 0–3 and CEAP 4–6. For those with venous pathology, scores for disease-specific and generic quality-of-life questionnaires improved without reaching statistical significance (Table 6). Those with C4-6 disease benefitted the most, with improvements in VDS score of 1, AVVQ of 6, and SF-12 of 10. The only statistically significant change was in VDS score from week 0 to 6 (p=0.03, Mann–Whitney *U*-test). After two weeks of NMES abstinence, the improvements in venous clinical scores were sustained. Healthy subjects, and those with mild clinical symptoms, saw no improvements in generic quality of life over 6 weeks.

**Table 6:** Mean Quality of Life Scores and Changes Over Time

	CEAP C0-3			CEAP C4-6		
	Week 0 N=17	Week 6 N=16	Week 8 N=11	Week 0 N=18	Week 6 N=14	Week 8 N=13
VCSS	2.9	2.9	3.4	9.6	6.5	7.4
VDS	0.69	0.71	0.78	1.9	1.1**	1.3
AVVQ	9.6	8.0	9.2	26.6	22.4	23.6
EQ5D	0.81	0.86	0.90	0.7	0.7	0.7
SF-12	99	102	94	85	95	87
CES-D	8.9	7.3	10.6	12.4	11.1	12.5

**Notes**: **p<0.01, Mann–Whitney U, week 0 to week 6.

**Abbreviations**: VCSS, venous clinical severity score; VDS, venous disability score; AVVQ, Aberdeen varicose vein questionnaire; SF-12, short form 12.

### Device Usage and Diary

Devices were activated for a patient-reported mean time of 5.56±1.6 hours per day, 5 days a week. Devices were well tolerated by subjects. Some chose to activate the devices during the day, others at night. No subjects reported use of the device interfering with their activities of daily living, or mobilising. Skin irritation occurred in five subjects, and the device was discontinued where further use worsened it. The mean device usage prior to cessation was 122.7 hours, for a mean of 4.9±1 hours per day. No link was identified between length of use and incidence of skin irritation.

## Discussion

Morbidity and mortality from chronic venous disease, deep vein thrombosis (DVT), venous thromboembolism, and post-thrombotic syndrome are significant.^1^,^23^ A device that enhances the peripheral circulation could aid thromboprophylaxis in healthy individuals, and may help manage symptoms in those with pre-existing venous disease.

This study has shown that the geko^TM^ NMES device can increase venous peak velocities in the healthy leg, which is concordant with existing literature.^15^,^24^ NMES may be useful in the management of stasis-related disease (eg venous thromboembolism), where the rapid transition of blood through the valves of the deep venous plexi of the leg is seen to be important.^25^ This study did not attempt to measure thrombosis rates in our population. Interestingly, the use of the device enhances the circulation in patients with chronic venous disease, even those with obstructive pathology. The benefits of repeated use include relief from leg oedema and possible improvements in the quality of life of those with clinically severe venous symptoms. Laser Doppler fluximetry is increased in the feet of subjects with venous disease, which may indicate that blood flow to the skin of the foot is enhanced. This may be very helpful in the management of venous ulcers, especially where compression is not tolerated or contra-indicated. The mechanism for an enhanced effect with repeated use has not been elucidated. The authors postulate that this may be due to synergistic local and systemic factors (eg muscle tone with exercise, neuroendocrine modulation, or functional modulation of capillary networks). Significant changes in the arm Laser Doppler fluximetry support the important roles that exercise and enhanced venous return have on the circulatory system and perfusion of skin.

There were large variations in age and BMI characteristics between groups, which may have affected activity levels, and therefore venous symptoms, over the course of six weeks. We did not control or stratify for gender, activity, adjuvant compression therapy, fluid intake or balance, or menstrual cycle in females, which may have affected our results. There was overlap in some subjects between deep and superficial disease, and this was not isolated or explored. We did not assess perforator competency in isolation (which has been shown to modify the haemodynamics of the leg) as part of our protocol.^26^ The effect of calf perforators on the response to calf muscle pump action has not been explored but may have had a detrimental effect on therapy when present. We did not covertly monitor adherence to the six week protocol, so are evaluating the effects of the device on an intention to treat basis. The correlation between reported and actual compliance is not known, therefore the effect of the device on all parameters, including side effects, may have been underestimated as a result. The stimulation intensity used by subjects was not recorded, chiefly because the settings vary according to placement on the leg and the relative distance of electrodes to the underlying nerve, which changes with joint position. Subjects may have found the device either more or less tenable over the trial period, and changing stimulation and muscle contraction intensity may underlie some of the haemodynamic effects. The trial was underpowered to fully evaluate quality of life changes from using the device. For this reason, we have not attempted to perform any health economic arguments. However, the changes in AVVQ, SF-12, and CES-D could be described as clinically significant. The effect size demonstrated here may be used in the future for the purposes of power calculation.

The non-invasive nature of transcutaneous stimulation means that some subjects remain recalcitrant to its effects. In the authors’ experience, inability to elicit dorsiflexion with NMES renders effective treatment impossible. In 23% of our subjects, all with severe venous disease and a larger than average BMI, maximum device stimulation was inadequate. If oedema is a contributing factor, prior limb elevation or compression bandaging may help. Otherwise, IPC and exercise programs may be viable alternatives.^7^,^27–29^ Skin irritation occurred in five subjects, which were treated with simple emollients. We have raised this issue with the manufacturer of the device, and other device adhesives are currently under investigation.

NMES enhances the peripheral venous circulation of both healthy subjects and those with chronic venous disease, and may be helpful in managing clinical symptoms. It may be a useful adjunct in the prevention of VTE, particularly in those patient groups at high risk, with contraindications to anticoagulation.

## Data Sharing Statement

Anonymised data sharing will be considered according to reasonable request, by contacting the corresponding author directly.

## References

[1] Beebe-Dimmer JL, Pfeifer JR, Engle JS, Schottenfeld D. The epidemiology of chronic venous insufficiency and varicose veins. *Ann Epidemiol*. 2005;15(3):175–184. doi:10.1016/j.annepidem.2004.05.015 15723761

[2] White RH. The epidemiology of venous thromboembolism. *Circulation*. 2003;107(23 Suppl 1):I4–8. doi:10.1161/01.CIR.0000078468.11849.66 12814979

[3] Callam MJ. Epidemiology of varicose veins. *Br J Surg*. 1994;81(2):167–173. doi:10.1002/bjs.1800810204 8156326

[4] Maurins U, Hoffmann BH, Losch C, Jockel KH, Rabe E, Pannier F. Distribution and prevalence of reflux in the superficial and deep venous system in the general population–results from the Bonn Vein Study, Germany. *J Vasc Surg*. 2008;48(3):680–687. doi:10.1016/j.jvs.2008.04.029 18586443

[5] Williams KJ, Ayekoloye O, Moore HM, Davies AH. The calf muscle pump revisited. *J Vasc Surg*. 2014;1–6. doi:10.1016/j.jvsv.2013.10.053 26993396

[6] Baker SR, Stacey MC, Jopp-McKay AG, Hoskin SE, Thompson PJ. Epidemiology of chronic venous ulcers. *Br J Surg*. 1991;78(7):864–867. doi:10.1002/bjs.1800780729 1873720

[7] Nelson EA, Mani R, Thomas K, Vowden K. Intermittent pneumatic compression for treating venous leg ulcers. *Cochrane Database Syst Rev*. 2011;2:CD001899–CD001899. 10.1002/14651858.CD001899.pub3 21328252

[8] Vowden K. The use of intermittent pneumatic compression in venous ulceration. *Br J Nurs*. 2001;10(8):491–509. doi:10.12968/bjon.2001.10.8.5312 12066041

[9] Comerota AJ. Intermittent pneumatic compression: physiologic and clinical basis to improve management of venous leg ulcers. *J Vasc Surg*. 2011;53(4):1121–1129. doi:10.1016/j.jvs.2010.08.059 21050701

[10] Silverstein MD, Heit JA, Mohr DN, Petterson TM, O’Fallon WM, Melton LJ 3rd. Trends in the incidence of deep vein thrombosis and pulmonary embolism: a 25-year population-based study. *Arch Intern Med*. 1998;158(6):585–593. doi:10.1001/archinte.158.6.585 9521222

[11] Prandoni P, Lensing AW, Cogo A, et al. The long-term clinical course of acute deep venous thrombosis. *Ann Intern Med*. 1996;125(1):1–7. doi:10.7326/0003-4819-125-1-199607010-00001 8644983

[12] Cohen AT, Agnelli G, Anderson FA, et al. Venous thromboembolism (VTE) in Europe. The number of VTE events and associated morbidity and mortality. *Thromb Haemost*. 2007;98(4):756–764. doi:10.1160/TH07-03-0212 17938798

[13] Goldhaber SZ, Fanikos J. Prevention of deep vein thrombosis and pulmonary embolism. *Circulation*. 2004;110(16):e445–e447. doi:10.1161/01.cir.0000145141.70264.c5 15492324

[14] Morris JK, Fincham BM. Intermittent pneumatic compression for venous thromboembolism prophylaxis in total knee arthroplasty. *Orthopedics*. 2012;35(12):e1716–21. doi:10.3928/01477447-20121120-15 23218627

[15] Williams KJ, Moore HM, Davies AH. Haemodynamic changes with the use of neuromuscular electrical stimulation compared to intermittent pneumatic compression. *Phlebology*. 2015;30(5):365–372. doi:10.1177/0268355514531255 24722790

[16] Williams KJ, Davies A. The use of a novel neuromuscular electrical stimulation device in peripheral vascular disease. *Int J Case Rep Images*. 2014;5(11):744–747. doi:10.5348/ijcri-201462-CS-10048

[17] Williams KJ, Ravikumar R, Babber A, Davies AH. Can neuromuscular stimulation relieve the symptoms of chronic venous disease, and improve quality of life? (abstract). *BJS*. 2015;102(S5):9.

[18] Ravikumar R, Williams KJ, Davies A. Potential clinical applications of electrical muscle stimulation in venous disease (abstract). *BJS*. 2015;102(S5):45.

[19] Anderson SI, Whatling P, Hudlicka O, Gosling P, Simms M, Brown MD. Chronic transcutaneous electrical stimulation of calf muscles improves functional capacity without inducing systemic inflammation in claudicants. *Eur J Vasc Endovasc Surg*. 2004;27(2):201–209. doi:10.1016/j.ejvs.2003.10.003 14718904

[20] Kaulesar Sukul DM, den Hoed PT, Johannes EJ, van Dolder R, Benda E. Direct and indirect methods for the quantification of leg volume: comparison between water displacement volumetry, the disk model method and the frustum sign model method, using the correlation coefficient and the limits of agreement. *J Biomed Eng*. 1993;15(6):477–480. doi:10.1016/0141-5425(93)90062-4 8277752

[21] Vasquez MA, Munschauer CE. Revised venous clinical severity score: a facile measurement of outcomes in venous disease. *Phlebology*. 2012;27(Suppl 1):119–129. doi:10.1258/phleb.2012.012S16 22312078

[22] Eaton WW, Muntaner C, Smith C, Tien A, Ybarra M. Center for Epidemiologic Studies Depression Scale: review and revision (CESD and CESD-R). In: Maruish M, editor. *The Use of Psychological Testing for Treatment Planning and Outcomes Assessment*. 3rd ed. Lawrence Erlbaum; 2004:363–377.

[23] Baldwin MJ, Moore HM, Rudarakanchana N, Gohel M, Davies AH. Post-thrombotic syndrome: a clinical review. *J Thromb Haemost*. 2013;11(5):795–805. doi:10.1111/jth.12180 23433231

[24] Tucker AT, Maass A, Bain DS, et al. Augmentation of venous, arterial and microvascular blood supply in the leg by isometric neuromuscular stimulation via the peroneal nerve. *Int J Angiol*. 2010;19(1):e31–7. doi:10.1055/s-0031-1278361 22477572 PMC2949997

[25] Nicolaides AN, Kakkar VV, Field ES, Renney JT. The origin of deep vein thrombosis: a venographic study. *Br J Radiol*. 1971;44(525):653–663. doi:10.1259/0007-1285-44-525-653 5569959

[26] Delis KT, Husmann M, Kalodiki E, Wolfe JH, Nicolaides AN. In situ hemodynamics of perforating veins in chronic venous insufficiency. *J Vasc Surg*. 2001;33(4):773–782. doi:10.1067/mva.2001.112707 11296331

[27] Kan YM, Delis KT. Hemodynamic effects of supervised calf muscle exercise in patients with venous leg ulceration: a prospective controlled study. *Arch Surg*. 2001;136(12):1364–1369. doi:10.1001/archsurg.136.12.1364 11735861

[28] Padberg FT Jr, Johnston MV, Sisto SA. Structured exercise improves calf muscle pump function in chronic venous insufficiency: a randomized trial. *J Vasc Surg*. 2004;39(1):79–87. doi:10.1016/j.jvs.2003.09.036 14718821

[29] Comerota A. Intermittent pneumatic compression improves healing of venous ulcers. *Vascular*. 2009;17:S82–S82.

